# Effects of Omega‐3 Supplementation in Sarcopenia After Bariatric Surgery: A Triple‐Blind Randomized Controlled Clinical Trial

**DOI:** 10.1002/jcsm.70321

**Published:** 2026-06-14

**Authors:** André Vicente Bigolin, Júlia Iaroseski, Randhall Bruce Carteri, Giovanna Severino Rodrigues, Rafaela de Andrade, Luiz Alberto De Carli, Elisa Loch Razzera, Izabele Vian, Roberta de Almeida da Silva, Antônio Nocchi Kalil, Luis Fernando Ferreira

**Affiliations:** ^1^ Santa Casa of Porto Alegre Hospital Porto Alegre Brazil; ^2^ Institute of Cardiology of Rio Grande do Sul/University Foundation of Cardiology Porto Alegre Brazil; ^3^ Federal University of Health Sciences of Porto Alegre Porto Alegre Brazil; ^4^ Queen's University of Belfast Belfast UK

**Keywords:** bariatric surgery, dietary supplements, muscle strength, omega‐3 fatty acids, sarcopenia

## Abstract

**Background:**

Bariatric surgery is an effective treatment for obesity but induces substantial muscle loss, potentially leading to sarcopenia. Omega‐3 fatty acids have been associated with improved muscle metabolism, but their effect in the postoperative period of bariatric surgery remains unclear.

**Methods:**

A triple‐blind randomized clinical trial following CONSORT guidelines. Patients undergoing bariatric surgery were randomly assigned to receive omega‐3 supplementation (2000 mg/day of EPA + DHA) or placebo (sunflower oil) for 90 days, including 15 days preoperatively and 75 days postoperatively. Assessments were performed at baseline, 45 days and 105 days after the start of supplementation. Outcomes included skeletal muscle mass (bioelectrical impedance [BIA]), handgrip strength (HGS) and functional performance (timed up and go [TUG] test).

**Results:**

Fifty‐eight patients were randomized (81% women; mean age 38.2 ± 9.9 years; mean body mass index [BMI]: 41.1 ± 4.6 kg/m^2^). At baseline, intervention and control groups were comparable in weight (112.7 ± 19.1 vs. 113.0 ± 17.9 kg; *p* = 0.946), muscle mass (33.5 ± 7.5 vs. 32.5 ± 7.3 kg; *p* = 0.603), HGS (33.3 ± 9.6 vs. 35.4 ± 10.2 kgf; *p* = 0.428) and TUG (8.7 ± 3.0 vs. 8.7 ± 1.8 s; *p* = 0.912). Between baseline and 105 days, muscle mass decreased significantly in both groups (−3.1 ± 0.3 kg; −2.2 ± 0.5 kg; both *p* < 0.001), without interaction between groups (*p* = 0.300). HGS remained stable (Δ −0.8 to +2.3 kgf; all *p* ≥ 0.364), and TUG performance did not change significantly (Δ 0.0–1.1 s; all *p* ≥ 0.106). Weight loss was marked in both groups (−19.3 ± 1.9 vs. −17.7 ± 2.1 kg; both *p* < 0.001). The incidence of gastrointestinal symptoms ≥ 3 times/week was similar (34.5% vs. 13.6% at 45 days; *p* = 0.114). No serious adverse events occurred.

**Conclusions:**

Omega‐3 supplementation (2000 mg/day) during the perioperative period of bariatric surgery did not prevent muscle mass loss nor improve muscle strength or functional performance within 105 days of follow‐up. Although the supplementation was safe and well tolerated, it did not demonstrate efficacy in mitigating early sarcopenia‐related outcomes in this population.

**Trial Registration:** ClinicalTrials.gov: NCT06494566

## 1 Introduction

Obesity is a highly prevalent chronic disease and has been associated with a reduction of up to 22% in total life expectancy [1]. Its systemic effects stem from excess adipose tissue infiltrating organs, triggering inflammatory and metabolic pathways that predispose to cardiovascular diseases. Unlike other tissues, fat infiltration also occurs in skeletal muscle [2]. This intramuscular fat may impair force production, with marked degradation of enzymes such as myostatin, NF‐κB and MuRF‐1 in individuals with severe obesity [3].

This metabolic impairment contributes to sarcopenic obesity, a condition characterized by reduced muscle protein synthesis in response to anabolic stimuli and increased protein degradation [4]. Combined with obesity, the diagnosis of sarcopenia, as established by the EWGSOP2 [5], relies primarily on muscle strength (probable sarcopenia), then on muscle mass (confirmation) and physical performance (severity), assessed by tests like the timed up and go (TUG) test, Short Physical Performance Battery (SPPB) or gait speed.

Although bariatric surgery is an effective intervention for obesity, postoperative weight loss has been accompanied by significant loss of muscle mass, largely driven by metabolic adaptation and reduced protein intake. The potential aggravation of sarcopenic obesity after surgery remains poorly understood, underscoring the need for strategies to prevent its metabolic consequences and mitigate weight regain [6].

Among potential therapeutic approaches, omega‐3 polyunsaturated fatty acids (PUFAs) have been shown to enhance skeletal muscle protein synthesis and attenuate saturated fat‐induced muscle atrophy [7]. Supplementation with PUFAs has yielded gains in muscle mass, strength and functional performance in populations predisposed to sarcopenia [8, 9, 10]. Nevertheless, the impact of omega‐3 supplementation in the postoperative period of bariatric surgery has not yet been investigated. Therefore, this study aimed to evaluate the effects of omega‐3 supplementation in patients undergoing bariatric surgery during the first 105 postoperative days.

## 2 Methods

### 2.1 Trial Design

This study is a triple‐blind randomized clinical trial conducted according to the CONSORT recommendation [11]. The study protocol is registered in ClinicalTrials.gov, under the number NCT06494566.

**FIGURE 1:**
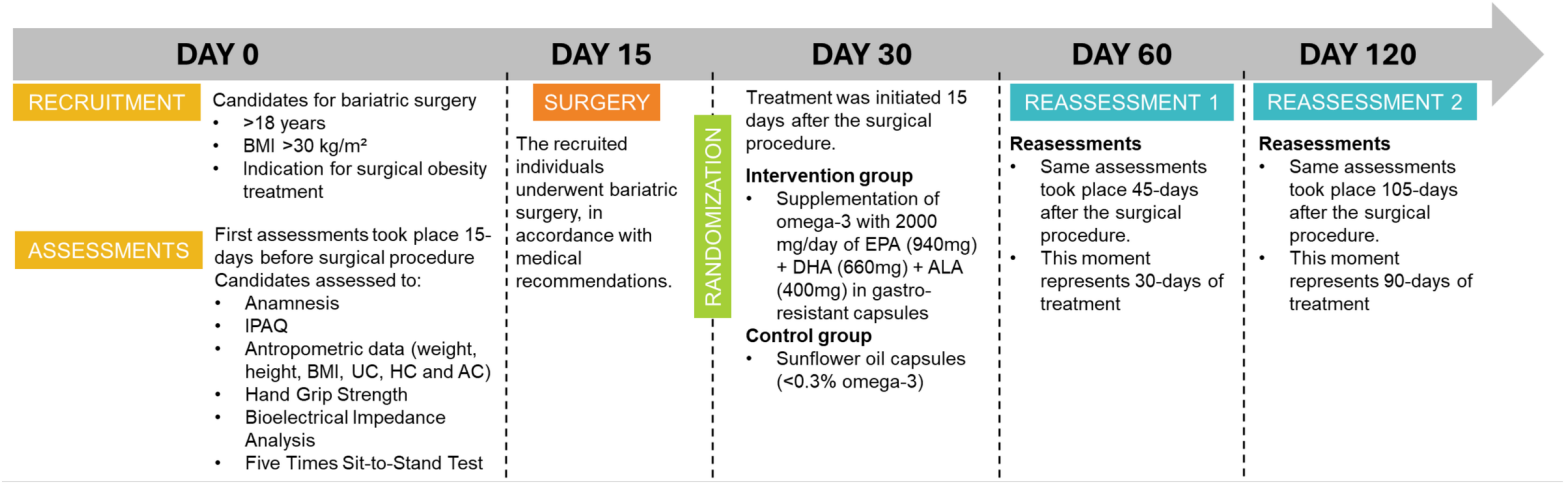
Study design. AC, arm circumference; BMI, body mass index (kg/m^2^); HC, hip circumference; IPAQ, International Physical Activity Questionnaire; UC, umbilical circumference.

Patients were evaluated 15 days before the surgical procedure (baseline–Day 0). No treatment was initiated at this time. Treatment (or placebo) was initiated 15 days (Day 30) after the surgical procedure and continued for 90 days (until Day 120). The first reassessment occurred 45 days after the surgical procedure (Day 60), and the last occurred 105 days after the surgical procedure (Day 120). Trial design can be seen in Figure 1.

### 2.2 Patient and Eligibility Criteria

Included were patients who were candidates for bariatric surgery, who were older than 18 years, had a BMI above 30 kg/m^2^ sought or were referred to the obesity surgical treatment outpatient clinic of a single centre. Excluded were those patients who used omega‐3, those who had a history of cardiac arrhythmia and those with contraindications to bioelectrical impedance analysis (BIA) or a TUG test.

### 2.3 Intervention and Comparator

Patients were randomly divided into two groups. The proposed intervention (Group 1) was dietary supplementation of omega‐3 with 2000 mg/day of EPA (940 mg) + DHA (660 mg) + ALA (400 mg) in gastro‐resistant capsules; and placebo with sunflower oil capsules (2000 mg) (< 0.3% omega‐3). Supplementation began 15 days after bariatric surgery and remained for 90 days. Participants were instructed to take four capsules of 500 mg each daily, two at lunch and two at dinner, for 90 days of use.

The control group (Group 2) received the same dosage of identical sunflower oil capsules (< 0.3% omega‐3) during the same time and following the same protocol adopted to intervention group.

### 2.4 Assessments and Outcomes

The primary outcome was sarcopenia, represented by its indicators, according to the EWGSOP2 [5]: muscle strength, skeletal muscle mass and physical performance.

The patients were evaluated preoperatively, 15 days before the surgical procedure. During the postoperative period, the patients were reassessed at two time points: 45 and 105 days after the surgical procedure.

At each assessment, participants underwent a standardized interview and physical evaluations as follows.

First, an adapted anamnesis was performed, when the patient was asked about the presence of gastrointestinal symptoms such as epigastralgia, nausea, heartburn, difficulty sleeping due to reflux symptoms, vomiting episodes and the need to use medication to treat such symptoms. The presence of such symptoms three or more times a week or the presence of vomiting was considered relevant.

To assess the physical activity level and/or the sedentary behaviour, the shortened version of the Physical Activity Questionnaire (IPAQ) was applied to all participants [12].

Weight and height were measured using a stadiometer with 0.01 m precision (Cescorf, Porto Alegre, Brazil), with the patient standing, barefoot and wearing minimal clothing. Weight and height were used to calculate the body mass index (BMI). Cut‐off points followed World Health Organization recommendations [13]. Other anthropometric data collected included the umbilical, hip and arm circumferences (UC, HC and AC, respectively).

Muscle strength was assessed by handgrip (Jamar hydraulic hand dynamometer—Sammons Preston Rolyan, IL, USA). The data are presented in kg of force. The patients' position and test realization followed the recommendations of the American Society of Hand Therapy [14].

Skeletal muscle mass and body composition were assessed by BIA (InBody 720 Fat Analyser, InBody CO, Seoul, South Korea). The patient's preparation and positioning followed those recommended by the European Society for Clinical Nutrition and Metabolism [15]. The data collected were the impedance resistance, and the muscle and fat mass were calculated using the formula proposed by Janssen et al. [16].

To assess physical performance, TUG test was performed, following the recommendation from Kear et al. [17].

### 2.5 Adherence to Intervention/Placebo

The adherence was assessed by capsule counts at each follow‐up visit. Based on the number of capsules returned, participants reported less than 10% of non‐covered days over the supplementation period, indicating high compliance with the prescribed regimen. Adherence rates were similar between the omega‐3 and placebo groups.

### 2.6 Harms

Potential harms and adverse events were prospectively defined and systematically assessed at all study visits. At each evaluation (baseline, 60 days and 120 days), participants were specifically asked about gastrointestinal symptoms, including epigastric pain, nausea, heartburn, reflux‐related nocturnal disturbances and vomiting episodes, as well as the need for pharmacological treatment of such symptoms. Events were considered clinically relevant when they occurred three or more times per week or when vomiting was reported. In addition, patients were monitored for systemic or serious adverse events, such as hepatotoxicity, cardiovascular complications (e.g., arrhythmias and thromboembolic events) and any clinically significant laboratory or clinical findings. Hospital readmissions, surgical complications attributable to supplementation and deaths were also predefined as serious adverse outcomes.

### 2.7 Sample Size

The sample size was determined using the study by Smith et al. [18] as a reference. HGS was considered the main outcome for this calculation, as it is the first criterion for defining sarcopenia, according to EWGSOP2 [5]. Considering an increase of 6.6% in HGS in the omega‐3 group, with a calculated effect size of 0.377, with an error of 5% and a confidence level of 85%, the total sample size was 66 patients, or 33 per group. Sample size calculation was performed using G‐Power software, Version 3.1.9.7 (Heinrich Heine University, Düsseldorf, Germany).

### 2.8 Randomization and Blinding

Through blocks of two participants, in the ratio of 1:1 for 2 treatment groups: (1) omega‐3 and (2) placebo. A block was used for every two participants who entered the study, resulting in a total of 75 blocks, or 150 patients. Randomization was performed with the online software random.org. The blocks were drawn at the beginning of the study, and the numbering of each block was stored in a non‐transparent, serialized and sealed envelope. The capsules were packaged in identical opaque vials by a single investigator who did not participate in data collection or analysis.

The triple‐blinding process was ensured by the following: Participants were unaware of the type of medication they were taking, whether omega‐3 capsules or placebo. Only one researcher, not involved in clinical data collection, had access to this information; outcome assessors were unaware of which group the participants were allocated to; and data analyses were performed by a researcher who was not involved at any other point in the research and performed the analyses using a dataset blinded to the clustering (clustering was referred to as ‘Group 1’ and ‘Group 2’ in the database).

### 2.9 Ethical Procedures

The project was approved by the Research Ethics Committee of the Institute of Cardiology, Porto Alegre, Brazil, under letter no. 3.240.104. Volunteers read and signed the Informed Consent Form. The entire research was conducted following Resolution 196/96 of the National Health Council (Brazil) and adhered to the principles of the Declaration of Helsinki for research involving human subjects. Data were processed in accordance with the General Data Protection Law (Brazilian Law No. 13.709/2018).

### 2.10 Statistical Methods

Continuous data are presented as mean and standard deviation, and categorical data as absolute and relative frequency. Data normality was tested using the Kolmogorov–Smirnov test. Baseline comparisons between groups were performed using Student's *t*‐test for independent samples. A 95% significance level (*p* < 0.05) was adopted for all analyses.

Longitudinal intra‐ and intergroup comparisons were performed using generalized estimating equations (GEE) to account for the correlation among repeated measurements over time. All outcomes analysed were continuous variables; therefore, GEE models with a normal distribution, identity link function and an unstructured working correlation matrix were applied to accommodate within‐subject dependence. Robust (sandwich) standard errors were used to ensure the reliability of the estimates. Each model included group (omega‐3 vs. placebo), time (baseline, 60 days and 120 days) and the prespecified group‐by‐time (ARM × TIME) interaction term, which was considered the primary effect of interest. When appropriate, multiple comparisons were adjusted using the Bonferroni method.

The standardized effect size (SES) was used to assess the magnitude of the intervention effect from raw means and standard deviations. According to Cohen (1988), effect sizes were classified as small (< 0.5), moderate (0.5–0.8) and large (> 0.8). All data were processed using SPSS V27.0 (IBM Statistics, Chicago, IL, USA).

The principle of intention to treat was applied to patients who, for some reason, dropped out of the study or did not complete the protocol.

## 3 Results

Eighty‐eight eligible patients were identified. Sixty‐two patients were enrolled. Trial enrolment was interrupted on one occasion because of the recommended discontinuation of care and elective surgery due to the COVID‐19 pandemic. This was the main reason for those who declined to participate.

**FIGURE 2:**
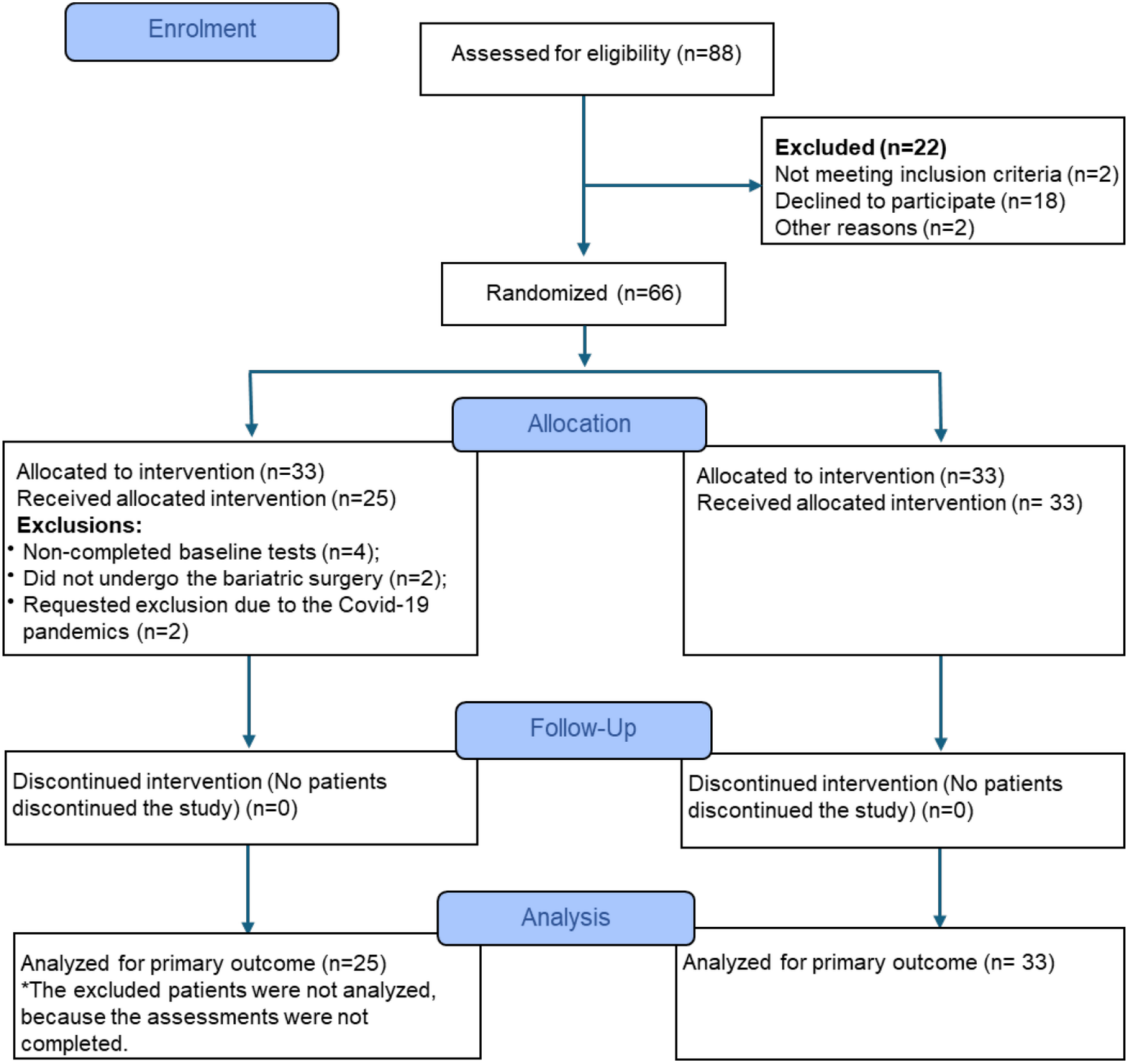
CONSORT inclusion flow chart.

Among the patients who met the inclusion criteria, four patients were excluded from the study because they had not completed the baseline test, two did not undergo the procedure, and two requested exclusions from the study. Fifty‐eight patients were randomized and analysed via the intention‐to‐treat model. The inclusion flow chart is shown in Figure 2.

**FIGURE 3:**
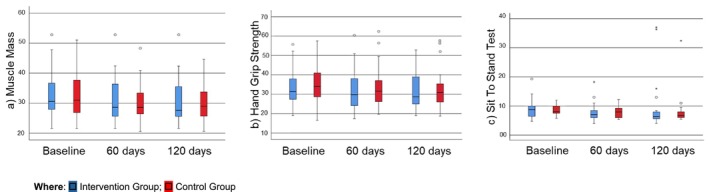
Characterizations by group over time: (a) muscle mass, (b) handgrip strength and (c) sit‐to‐stand test. *Note:* 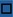, intervention group; 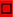, control group.

The sample consisted predominantly of women (81%). The mean age was 38.2 years. The most commonly performed technique was the gastric bypass technique. The characteristics of the samples, as well as the comparisons between the groups, can be seen in Table 1 and in Figure 3.

**TABLE 1:** Sample baseline characterization (*n* = 58).

Variables	Intervention group (*n* = 33)	Control group (*n* = 25)	*p*
Age (years)^a^	38.5 ± 9.4	38.0 ± 10.8	0.868
Weight (kg)^a^	112.7 ± 19.1	113.0 ± 17.9	0.946
Height (cm)^a^	167.1 ± 8.6	164.3 ± 9.8	0.258
Visceral fat (kg)^a^	25.9 ± 4.2	26.2 ± 3.9	0.791
Fat mass (kg)^a^	53.4 ± 10.6	55.4 ± 11.5	0.504
Muscle mass (kg)^a^	33.5 ± 7.5	32.5 ± 7.3	0.603
Body fat percentage (%)^a^	47.6 ± 5.6	49.2 ± 6.2	0.310
Body mass index (kg/m^2^)^a^	40.3 ± 4.3	41.9 ± 4.8	0.189
Hip circumference (cm)^a^	129.0 ± 9.9	132.2 ± 10.8	0.245
Arm circumference (cm)^a^	36.8 ± 3.7	37.1 ± 2.8	0.735
Umbilical circumference (cm)^a^	115.2 ± 14.9	118.0 ± 11.1	0436
HGS (kgf)^a^	33.3 ± 9.6	35.4 ± 10.2	0.428
TUG test (s)^a^	8.7 ± 3.0	8.7 ± 1.8	0.912
Surgery: Bypass^b^	18 (54.5)	14 (56.0)	1.000
Sex: Female^b^	26 (78.8)	21 (84.0)	0.742
Comorbidities^b^			
Hypertension	10 (30.3)	8 (32.0)	1.000
Hepatic steatosis	13 (39.4)	9 (36.0)	1.000
Arthropathy	9 (27.3)	6 (24.0)	1.000
Dyslipidaemia	8 (24.2)	5 (20.0)	0.760
SAHOS	4 (12.1)	1 (4.0)	0.378
DM2	3 (9.1)	5 (20.0)	0.272

*Note:* p‐value: Student's *t*‐test for independent samples.

Abbreviations: %: body weight percentage; DM2, diabetes mellitus Type 2; HGS, hand grip strength; SAHOS, Obstructive Sleep Apnea and Hypopnea Syndrome; TUG, timed up and go test.

^a^ Continuous data, presented in mean and standard deviation.

^b^ Categoric data, presented in absolute and relative sample.

Table 2 presents the analysis of the variables considering the interaction between moment groups. Despite a reduction in averages overtime for all the variables analysed, none of the interactions over time were statistically significant. The delta‐differences between moments can be found in Table 3.

**TABLE 2:** Model‐based adjusted means and group‐by‐time interaction estimates from generalized estimating equations (*n* = 58).

Variables	Intervention group (*n* = 33)		Control group (*n* = 25)		p‐interaction	Effect size^a^
	M (SE)	CI (lower–upper)	M (SE)	CI (lower–upper)		
Weight (kg)						
Baseline	112.7 (3.3)	106.3–119.1	113.0 (3.5)	106.1–119.9	0.761	0.085
60 days	99.5 (3.1)	93.3–105.6	101.2 (3.4)	94.5–108.0		
120 days	93.3 (3.3)	86.7–99.9	95.3 (3.4)	88.7–101.9		
Visceral fat (kg)						
Baseline	25.9 (0.7)	24.5–27.4	26.1 (0.7)	24.6–27.6	0.654	0.025
60 days	22.1 (0.8)	20.5–23.6	22.4 (0.9)	20.5–24.2		
120 days	19.4 (0.9)	17.6–21.3	20.2 (1.0)	18.2–22.2		
Fat mass (kg)						
Baseline	53.4 (1.8)	49.9–57.0	55.4 (2.2)	51.0–59.8	1.000	0.120
60 days	44.1 (1.8)	40.7–47.6	46.1 (2.5)	41.1–51.1		
120 days	38.5 (2.0)	34.6–42.4	40.5 (2.6)	35.3–45.7		
Muscle mass (kg)						
Baseline	33.5 (1.3)	31.0–36.0	32.5 (1.4)	29.7–35.2	0.300	0.003
60 days	30.8 (1.2)	28.4–33.3	30.4 (1.3)	27.8–33.0		
120 days	30.4 (1.2)	28.0–32.8	30.2 (1.3)	27.7–32.8		
Body fat percentage (%)						
Baseline	47.6 (1.0)	45.8–49.5	49.2 (1.2)	46.9–51.6	0.845	0.120
60 days	44.0 (1.2)	41.7–46.3	45.1 (1.5)	42.2–48.1		
120 days	40.5 (1.4)	37.8–43.2	41.7 (1.7)	38.4–45.0		
Body mass index (kg/m^2^)						
Baseline	40.3 (0.7)	38.9–41.8	41.9 (0.9)	40.1–43.8	0.956	0.344
60 days	35.3 (0.8)	33.8–36.8	37.1 (0.9)	35.2–38.9		
120 days	33.0 (0.8)	31.3–34.6	34.7 (1.0)	32.8–36.6		
Hip circumference (cm)^a^						
Baseline	129.0 (1.7)	125.7–132.4	132.2 (2.1)	128.1–136.4	0.500	0.289
60 days	118.9 (1.6)	115.7–122.1	123.6 (2.2)	119.2–128.0		
120 days	115.1 (1.8)	115.6–118.7	116.6 (2.5)	111.8–121.5		
Arm circumference (cm)^a^						
Baseline	36.8 (0.6)	35.6–38.1	37.1 (0.5)	36.0–38.2	0.493	0.125
60 days	34.0 (0.6)	32.8–35.3	34.7 (0.6)	33.5–35.8		
120 days	32.9 (0.7)	31.5–34.3	32.9 (0.7)	31.6–34.3		
Umbilical circumference (cm)^a^						
Baseline	115.2 (2.5)	110.2–120.2	118.0 (2.2)	113.7–122.2	0.221	0132
60 days	104.2 (2.4)	99.4–108.8	108.8 (2.3)	104.3–113.3		
120 days	100.0 (2.4)	95.3–104.8	102.6 (2.1)	98.5–106.8		
HGS (kgf)						
Baseline	33.3 (1.6)	30.1–36.5	35.4 (2.0)	31.5–39.3	0.734	0.255
60 days	32.4 (1.8)	28.9–36.0	33.2 (2.0)	29.3–37.2		
120 days	32.5 (1.7)	29.2–35.7	33.1 (1.9)	29.3–36.9		
TUG test (s)						
Baseline	8.7 (0.5)	7.7–9.8	8.7 (0.3)	8.0–9.4	0.719	0.012
60 days	7.6 (0.4)	6.7–8.5	7.9 (0.4)	7.2–8.7		
120 days	8.7 (1.3)	6.1–11.2	8.1 (1.0)	6.1–10.1		

*Note:* Values are adjusted marginal means (M) with standard errors (SE) and 95% confidence intervals derived from generalized estimating equations (GEE) models, with Bonferroni adjustment.

Abbreviations: %, body weight percentage; HGS, hand grip strength; TUG, timed up and go test.

^a^ By Cohen's *d*.

**TABLE 3:** Model‐based adjusted mean differences between time points derived from generalized estimating equations (*n* = 58).

Variables	Intervention (*n* = 33)		*p*	Control (*n* = 25)		*p*
	M (SE)	CI (lower–upper)		M (SE)	CI (lower–upper)	
Weight (kg)						
Baseline–60 days	13.2 (1.4)	9.8–16.6	**< 0.001**	11.8 (1.4)	8.4–15.1	**< 0.001**
60–120 days	6.1 (0.9)	4.1–8.2	**< 0.001**	5.9 (1.0)	3.6–8.3	**< 0.001**
Baseline–120 days	19.3 (1.9)	14.8–23.9	**< 0.001**	17.7 (2.1)	12.5–22.9	**< 0.001**
Visceral fat (kg)						
Baseline–60 days	3.9 (0.5)	2.7–5.0	**< 0.001**	3.8 (0.5)	2.6–4.9	**< 0.001**
60–120 days	2.6 (0.4)	1.7–3.5	**< 0.001**	2.1 (0.4)	1.2–3.0	**< 0.001**
Baseline–120 days	6.5 (0.7)	4.9–8.1	**< 0.001**	5.9 (0.7)	4.2–7.7	**< 0.001**
Fat mass (kg)						
Baseline–60 days	9.3 (1.1)	6.6–12.0	**< 0.001**	9.3 (1.1)	6.7–11.9	**< 0.001**
60–120 days	5.6 (0.8)	3.7–7.5	**< 0.001**	5.6 (1.1)	3.0–8.1	**< 0.001**
Baseline–120 days	14.9 (1.6)	11.2–18.7	**< 0.001**	14.9 (1.8)	10.6–19.2	**< 0.001**
Muscle mass (kg)						
Baseline–60 days	2.6 (0.3)	1.9–3.4	**< 0.001**	2.1 (0.4)	1.1–3.0	**< 0.001**
60–120 days	0.4 (0.2)	−0.0‐0.9	0.570	0.1 (0.2)	−0.4–0.7	1.000
Baseline–120 days	3.1 (0.3)	2.3–3.9	**< 0.001**	2.2 (0.5)	1.1–3.3	**< 0.001**
Body fat percentage (%)						
Baseline–60 days	3.6 (0.5)	2.4–4.9	**< 0.001**	4.1 (0.6)	2.7–5.5	**< 0.001**
60–120 days	3.5 (0.4)	2.4–4.6	**< 0.001**	3.5 (0.6)	2.0–5.0	**< 0.001**
Baseline–120 days	7.2 (0.8)	5.2–9.1	**< 0.001**	7.6 (0.8)	5.5–9.6	**< 0.001**
Body mass index (kg/m^2^)						
Baseline–60 days	5.0 (0.5)	3.9–6.1	**< 0.001**	4.9 (0.5)	3.7–6.0	**< 0.001**
60–120 days	2.3 (0.3)	1.6–3.0	**< 0.001**	2.3 (0.3)	1.6 (3.1)	**< 0.001**
Baseline–120 days	7.3 (0.6)	5.9–8.8	**< 0.001**	7.2 (0.7)	5.6–8.8	**< 0.001**
Hip circumference (cm)^a^						
Baseline–60 days	10.1 (1.1)	7.5–12.7	**< 0.001**	8.6–1.4	5.2–12.0	**< 0.001**
60–120 days	3.8 (0.7)	2.1–5.5	**< 0.001**	7.0 (1.1)	4.3–9.6	**< 0.001**
Baseline–120 days	13.9 (1.2)	10.9–16.9	**< 0.001**	15.6 (1.6)	11.7–19.5	**< 0.001**
Arm circumference (cm)^a^						
Baseline–60 days	2.8 (0.4)	1.8–3.8	**< 0.001**	2.4 (0.5)	1.1–3.7	**< 0.001**
60–120 days	1.1 (0.4)	0.1–2.0	**< 0.001**	1.7 (0.4)	0.8–2.7	**< 0.001**
Baseline–120 days	3.9 (0.5)	2.7–5.1	**< 0.001**	4.2 (0.6)	2.6–5.7	**< 0.001**
Umbilical circumference (cm)^a^						
Baseline–60 days	11.0 (1.3)	8.0–14.0	**< 0.001**	9.2 (1.4)	5.8–12.5	**< 0.001**
60–120 days	4.2 (1.0)	1.8–6.5	**< 0.001**	6.2 (1.3)	3.1–9.3	**< 0.001**
Baseline–120 days	15.2 (1.7)	11.0–19.3	**< 0.001**	15.4 (2.0)	10.5–20.2	**< 0.001**
HGS (kgf)						
Baseline–60 days	0.9 (0.9)	−1.3–3.1	1.000	2.1 (1.4)	−1.2–5.5	0.364
60–120 days	−0.0 (0.6)	−1.6–1.5	1.000	0.1 (0.4)	−1.0–1.2	1.000
Baseline–120 days	0.8 (1.1)	−1.7–3.4	1.000	2.3 (1.5)	−1.3–5.8	0.377
TUG test (s)						
Baseline–60 days	1.1 (0.4)	0.2–2.0	0.900	0.7 (0.3)	−0.1–1.5	0.106
60–120 days	−1.1 (1.3)	−4.2–2.1	1.000	−0.1 (1.1)	−2.8–2.6	1.000
Baseline–120 days	0.0 (1.2)	−2.7–2.8	1.000	0.6 (1.0)	−1.8 (3.0)	1.000

*Note:* Mean differences represent model‐based estimates derived from generalized estimating equations (GEE), expressed as adjusted differences between time points with standard errors (SE) and 95% confidence intervals; In bold: difference statistically significant.

Abbreviations: %, body weight percentage; HGS, hand grip strength; M, median; p‐interaction, generalized estimating equations (GEE) model, with Bonferroni adjustment; SE, standard error; TUG, timed up and go test.

^a^ By Cohen's *d*.

The difference in means was statistically significant for all variables and moments evaluated, except for muscle mass, when 60 days were compared with 120 days (intervention *p* = 0.057; control *p* = 1.000), as were HGS and TUG, which did not present differences in means at any of the moments evaluated, both in the intervention group and in the control group, as shown in Table 3. The main outcomes of sarcopenia, muscle mass, HGS and TUG score are shown in the charts in Figure 2.

**FIGURE 4:**
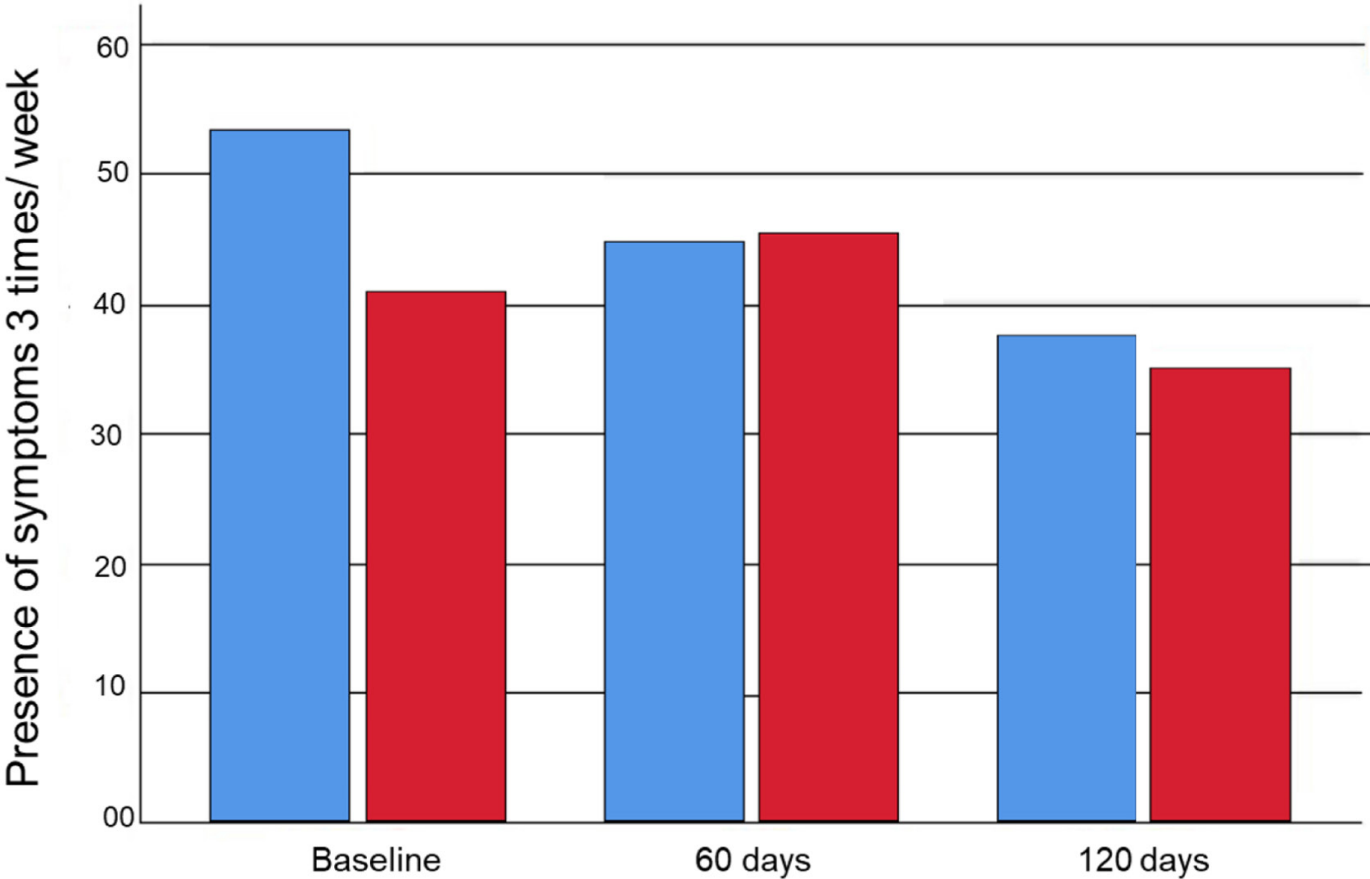
Presence of any gastrointestinal symptoms at baseline, 60 days and 120 days. *Note:* 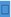, intervention group; 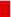, control group.

The incidence of gastrointestinal symptoms occurring three or more times per week was similar between groups (Figure 4). No serious adverse events occurred during the study period in either group. Adverse events were predominantly mild gastrointestinal symptoms. The proportion of participants reporting any adverse event was similar between the omega‐3 and placebo groups, and no statistically significant between‐group differences were observed. No participant discontinued supplementation due to adverse events or intolerance.

A summary of delta differences, as a secondary analysis, was explored and can be accessed in Appendix A of this study; and the results for physical activity level and/or the sedentary behaviour, assessed by IPAQ, can be found in Appendix B. Blood‐based biomarkers, including HbA1c, NT‐proBNP and C‐reactive protein, were assessed and showed no clinically relevant or statistically significant between‐group differences over the study period (data not shown).

## 4 Discussion

Obesity treatment has remained challenging, with postoperative weight regain continuing to be a major concern after bariatric surgery. Preservation of muscle mass is crucial for metabolic activity and for optimizing outcomes in weight control and comorbidities such as diabetes [19, 20].

Sarcopenia, although commonly studied in older adults, also occurs in patients with various chronic conditions [21]. One key mechanism involves inflammatory cascades that impair muscle synthesis. Omega‐3 PUFAs have been shown to possess anti‐inflammatory properties, potentially mitigating sarcopenia [22]. Most studies focus on EPA (C20:5 n‐3) and DHA (C22:6 n‐3), abundant in fish, which have been linked to cardiovascular benefits due to their anti‐inflammatory effects [23].

Diagnostic suspicion of sarcopenia is based primarily on loss of strength [5]. In this study, preoperative handgrip strength (HGS) was similar between groups (Group 1: mean 33.3 kgf, SD 1.64; Group 2: mean 35.4 kgf, SD 2.0). In the literature, healthy women have a mean HGS of 33.7 ± 7.1 kgf, whereas men tend to have higher values (53 kgf), which are also influenced by age and body weight^9^.

HGS remained stable throughout the postoperative period, with no significant differences between time points or groups. These findings are corroborated by Coral et al. [24], who reported stability in HGS over 6 months after bariatric surgery in a similar population.

The literature also indicates that 6 months after bariatric surgery, patients show improvements in performance tests (gait speed and TUG) compared with preoperative measurements, despite reductions in muscle mass [24]. This may be attributed to increased agility due to weight loss and improved muscle quality. In our study, TUG scores did not differ significantly between time points. Obese patients have greater muscle mass, but this tissue exhibits high fatty infiltration. Postoperative fat loss may improve muscle quality, supporting strength and functional performance despite reduced muscle quantity [25].

Compared with placebo, omega‐3 supplementation did not affect the evaluated variables, differing from Smith et al. [18], who reported increases in muscle strength and volume with supplementation. Other RCTs, such as Logan et al. [26], and meta‐analyses, like the one by Huang et al. [27], corroborate these effects, although these studies were conducted in older populations. Patients undergoing bariatric surgery differ not only in age but also in other characteristics, like highest rates of muscle strength (due to muscle morphology and innervation), neurologic aspects impacting motor pathways and movement patterns, among others [28, 29, 30].

Although sarcopenia in adult populations is often linked to factors such as obesity, metabolic syndrome, physical inactivity, inadequate nutrition, vitamin D deficiency, endocrinopathies, gut microbiota imbalance, neuromuscular diseases, organ failure, malignancy and inflammatory disorders [29], in the older populations, it is primarily age‐related, driven by progressive muscle loss, chronic inflammation, hormonal changes, reduced physical activity and inadequate nutrition [28, 30].

During the preoperative period, higher muscle mass relative to body fat is observed, along with impairments in physical performance tests. Postoperatively, catabolic processes result from metabolic responses to surgical trauma and significant caloric restriction, influencing muscle metabolism [31]. Muscle mass decreased significantly between time points, unlike HGS and STS. This apparent paradox has complicated the diagnosis of sarcopenia in severely obese patients undergoing weight loss [32].

Patients were instructed to use supplementation for 90 days, a period of substantial muscle loss after bariatric surgery. Longer supplementation may be necessary to achieve measurable benefits [18]. Also, the prescribed dosage is still not well described. Whereas Bosco et al. [33] proposed usage similar to ours, Smith et al. [18] proposed a higher dose of 1.86 g EPA (20:5 n–3) and 1.50 g DHA (22:6 n–3)/d, which is equivalent to the n–3 PUFA content of 200–400 g freshwater fatty fish [34]. This study (four capsules daily) was based on Bosco et al. [33] and was well tolerated, with no increased gastrointestinal symptoms compared with placebo. Compared with placebo patients, the intervention group did not have greater gastrointestinal symptoms. This result demonstrates that supplementation can be well tolerated in the early postoperative period of bariatric surgery.

We acknowledge that this RCT has some limitations. For example, no distinction was made between surgical techniques during patient selection. Both Roux‐en‐Y gastric bypass and sleeve gastrectomy patients were included. Although the distribution of surgical procedures was similar between groups, the study aimed to evaluate the impact of surgically induced weight loss on muscle‐related outcomes. Diet, supplementation and nutritional guidance were consistent across participants, yet the absorptive differences inherent to gastric bypass could potentially affect omega‐3 or protein uptake, influencing body composition outcomes [35, 36].

Also, patient dropouts and non‐participation, possibly influenced by the post‐COVID‐19 context, may have affected follow‐up assessments. To mitigate this, analyses were performed according to the intention‐to‐treat principle.

The study was designed as a sequential clinical trial. The first interim analysis was conducted once over 40% of the planned sample had completed the evaluation period. As HGS, the primary endpoint, did not show meaningful changes within the first 105 days postoperatively, further enrolment was halted. Sequential trial designs offer the ethical advantage of minimizing patient exposure when no clear benefit is observed [37].

Despite these limitations, this study provides a reference for future investigations. Further research is needed to define and diagnose sarcopenia following bariatric surgery more precisely, to evaluate longer‐term omega‐3 supplementation and to explore the influence of individualized surgical techniques on muscle‐related outcomes.

It is also important to highlight the fact that this was the first study in which omega‐3 fatty acids were used to evaluate outcomes in relation to the diagnosis of sarcopenia after bariatric surgery. In conclusion, in this RCT, omega‐3 supplementation in the perioperative period of bariatric surgery was not effective in preventing muscle mass loss, nor in promoting improvements in muscle strength or physical performance over 90 days of treatment. Despite the significant weight and muscle mass loss observed (which was expected), this occurred in both groups, regardless of omega‐3 use; HGS and performance on the TUG test remained stable, with no differences between the intervention and placebo groups.

### 4.1 Protocol and Statistical Analysis Plan

The project for this article can be found at clinicaltrials.gov, searching by the registration number.

## Funding

The authors have nothing to report.

## Conflicts of Interest

The authors declare no conflicts of interest.

## Data Availability

The additional data, which are not presented in the original paper, can be shared upon reasonable request to the corresponding author.

## Appendix A

Table A1 presents the adjusted between‐group contrasts at the primary timepoint (120 days) derived from the GEE models, along with the raw mean changes from baseline to 120 days for each group. No statistically significant differences between the omega‐3 and placebo groups were observed for any outcome at 120 days, and the magnitude of the adjusted contrasts was small across all variables, indicating a lack of clinically meaningful treatment effect.

**TABLE A1:** Adjusted between‐group contrasts at the primary timepoint (105 days) and raw mean changes (baseline to 105 days).

Outcome	Adjusted between‐group contrast at 120 days^a^ (omega‐3 × placebo)	95% CI	Raw mean change omega‐3 (baseline × 120 days)	Raw mean change placebo (baseline × 120 days)
Weight (kg)	−2.0	−7.1–3.1	−19.3	−17.7
Visceral fat (kg)	−0.8	−3.0–1.4	−6.5	−5.9
Fat mass (kg)	−2.0	−7.0–3.0	−14.9	−14.9
Muscle mass (kg)	0.2	−2.1–2.5	−3.1	−2.2
Body fat (%)	−1.2	−5.3–2.9	−7.2	−7.6
BMI (kg/m^2^)	−1.7	−4.2–0.8	−7.3	−7.2
Hip circumference (cm)	−1.5	−6.8–3.8	−13.9	−15.6
Arm circumference (cm)	0.0	−2.0–2.0	−3.9	−4.2
Umbilical circumference (cm)	−2.6	−8.0–2.8	−15.2	−15.4
Handgrip strength (kgf)	−0.6	−4.2–3.0	−0.8	+2.3

^a^ Adjusted contrasts derived from generalized estimating equations (GEE) models with group, time and group‐by‐time interaction using an identity link function, unstructured working correlation matrix and robust (sandwich) standard errors. Raw mean changes represent unadjusted differences between baseline and 120 days.

## Appendix B

To assess the physical activity level and/or the sedentary behaviour, the shortened version of the Physical Activity Questionnaire (IPAQ) was applied to all participants [12].

**Table:** **TABLE A2**   |   Model‐based adjusted means and group‐by‐time interaction estimates from generalized estimating equations for International Physical Activity Questionnaire (***n*** = 58).

IPAQ results	Intervention group (*n* = 33)		Control group (*n* = 25)		p‐interaction
	*n*	%	*n*	%	
Baseline					
Light	17	51.5	13	52.0	1.000
Moderate/high	16	48.5	12	48.0	
60 days					
Light	10	34.5	3	13.6	0.114
Moderate/high	19	65.5	19	86.4	
120 days					
Light	8	33.3	3	15.0	0.294
Moderate/high	16	66.7	17	85.0	

Abbreviations: %, relative sample; IPAQ, International Physical Activity Questionnaire; *n*, absolute value; p‐interaction, generalized estimating equations (GEE) models, with Bonferroni adjustment.
